# Post-traumatic stress disorder in crack/cocaine users

**DOI:** 10.1192/j.eurpsy.2024.839

**Published:** 2024-08-27

**Authors:** N. Ait Bensaid, Y. Bensalah, M. Sabir, F. El Omari

**Affiliations:** Psychiatric hospital ArRazi, salé, Morocco

## Abstract

**Introduction:**

Cocaine use has become popular in the form of crack and has spread throughout the world. Crack/cocaine use is often linked to serious social and psychiatric disorders, including post-traumatic stress disorder, and users appear to be at increased risk of physical and mental illness and social harm.

**Objectives:**

To determine the prevalence of post-traumatic stress disorder in patients followed and hospitalized in the addictology department at the Arrazi psychiatric hospital in Salé for management of crack/cocaine use disorder.

**Methods:**

This is a descriptive cross-sectional study using a questionnaire including sociodemographic and clinical criteria and a post-traumatic stress scale (PCLS) to investigate the existence of post-traumatic stress disorder in patients monitored and hospitalized for crack/cocaine use disorder in the addictology department at the Arrazi psychiatric hospital in Salé.

**Results:**

We collected 77 participants. The majority of patients were born in the city The average age of the participants was 27, with a male predominance (67%). The majority were unemployed at the time of the study, single, separated or divorced. For more than 50%, the start of drug use was more than 4 years ago. The smoked route (crack) is the most predominant, followed by the inhaled route and 1% for the injectable route. Some 37% were hospitalized in an addictology unit. Almost 65% of participants had a history of post-traumatic stress disorder.

**Conclusions:**

Co-morbidity between crack/cocaine use disorder and post-traumatic stress disorder is frequent among patients monitored and hospitalized in the addictology department at the Arrazi psychiatric hospital in Salé. There seems to be a need to develop new therapeutic strategies and to adapt existing programs to patients’ needs. In addition, understanding the profiles of patients suffering from this comorbidity in mental health facilities could help clinical staff to better accept their problems and behaviours, thus promoting treatment adherence and better outcomes.

**Disclosure of Interest:**

None Declared

